# Multiple Recessions Coverage Using the Modified Tunnel Technique and Connective Tissue Graft with or Without Cross-Linked Hyaluronic Acid: 2-Year Outcomes of RCT

**DOI:** 10.3390/jfb16030087

**Published:** 2025-03-04

**Authors:** Bartłomiej Górski, Izabela Maria Skierska, Andrea Gelemanović, Marija Roguljić, Darko Bozic

**Affiliations:** 1 Department of Periodontal and Oral Mucosa Diseases, Medical University of Warsaw, 02-097 Warsaw, Poland; 2 Mediterranean Institute for Life Sciences, University of Split, Šetalište Ivana Meštrovića 45, 21000 Split, Croatia; 3 Department of Periodontology, School of Medicine, University of Split, Šoltanska ulica 2A, 21000 Split, Croatia; 4 Department of Periodontology, School of Dental Medicine, University of Zagreb, Gunduliceva 5, 10000 Zagreb, Croatia; bozic@sfzg.hr

**Keywords:** connective tissue graft, gingival recession, hyaluronic acid, tunnel technique

## Abstract

There is continuing interest in using biologics in root coverage procedures. The aim of the present study was to explore the 2-year outcomes following multiple gingival recessions (GRs) coverage using the application of cross-linked hyaluronic acid (HA) in combination with modified coronally advanced tunnel (MCAT) together with subepithelial connective tissue graft (SCTG). Adopting a split-mouth design, 266 GRs were randomly allocated to either a test (MCAT + SCTG + HA) or control group (MCAT + SCTG). The main outcome variable was the stability of the obtained mean root coverage from 6 months to 24 months. Twenty-four patients were evaluated at the 2-year follow-up. Comparisons between test and control sides at the same time points were evaluated using the *t*-test for independent variables. The changes in time were compared by one-way analysis of variance with the Tukey post hoc test separately for the test and control groups. The study protocol was registered at ClinicalTrials.gov (NCT05045586). At 2 years, around ninety percent of recessions showed complete root coverage (87.02% of the test group and 91.90% of the control group). Mean root coverage did not differ between the two sides, with 81.37 ± 37.17% (test) and 84.63 ± 35.33% (control), respectively. Significant improvements in the reduction of gingival recession height, clinical attachment level gain, gingival thickness increase, and the root esthetic score were found in both groups after 2 years, but no statistically significant difference was observed between the groups. The adjunctive application of HA significantly improved soft tissue texture (STT, 0.94 ± 0.23 for the test group vs. 0.71 ± 0.46 for the control group). Treatment of multiple gingival recessions with MCAT + SCTG with or without HA yielded marked and comparable 2-year clinical outcomes, which could be maintained over a period of 24 months. The clinical relevance of the demonstrated significant difference in STT between groups may be minimal.

## 1. Introduction

Gingival recession (GR) is defined as an apical shift of the gingival margin with respect to the cemento–enamel junction (CEJ) with the exposure of a root surface [1]. GR occurs frequently in populations irrespective of oral hygiene standards and has a tendency to increase with age [2]. A recent systematic review and meta-analysis revealed that more than two-thirds of the population worldwide was affected by GR and the overall prevalence was 78.16% [3]. The etiology of GR is multifactorial, with one type being associated with anatomical factors (thin periodontal phenotype, tooth position) and another type with physiological or pathological factors (improper toothbrushing, gingivitis, cervical restorative margins, orthodontic treatment) [4]. The presence of recession is esthetically unacceptable and may lead to dentin hypersensitivity, as well as to carious and non-carious cervical lesions (NCCL) [5]. Cairo et al. [6] defined three types of GR on the basis of the level of interproximal clinical attachment level (CAL). In recession type 1 (RT1), there is no interproximal CAL; in recession type 2 (RT2), the loss of interproximal CAL is equal to or smaller than the buccal CAL loss; and in recession type 3 (RT3), the interproximal CAL is larger than the buccal CAL. In RT1, complete root coverage can be achieved; in RT2, complete root coverage can be predicted in some cases, but interdental CAL loss may limit GR reduction. In RT3, full root coverage is not achievable.

Several systematic reviews and meta-analyses have evaluated various techniques for the surgical treatment of GR [7]. The modified coronally advanced tunnel technique (MCAT) presents minimally invasive flap design [8]. This technique does not require vertical releasing incisions and leaves the interdental papillae intact, all of which improves blood supply preservation, aimed at overcoming the limitations of traditional approaches. MCAT demonstrated mean root coverage (MRC) of 82.75 ± 19.7% for single GR and 87.87 ± 16.45% for multiple GRs [9]. In adults with multiple gingival recessions, MCAT showed similar primary and secondary outcomes when compared to the coronally advanced flap (CAF) technique [10]. The use of subepithelial connective tissue graft (SCTG) may be considered as a gold standard, especially in cases in which both root coverage and gain of keratinized tissue (KTW) are required [11].

Hyaluronic acid (HA) is an endogenous polymer composed of disaccharide repeating sequences that form a carbohydrate polymer through alternating *β*-(1→4) and *β*-(1→3) glycosidic linkages between D-glucuronic acid and N-acetyl-D-glucosamine, which is biodegradable, biocompatible, and nontoxic [12]. HA is a vital element of the extracellular matrix, comprising a mechanical setting for cells and mediating the biochemical pathways required for tissue homeostasis. Based on its positive effect on wound healing, it is commonly used as a therapeutic in a wide range of applications in regenerative medicine. HA has been reported to boost inflammatory cell migration and proliferation, proinflammatory cytokine production, stabilization of the granulation matrix, and induce angiogenesis [13]. Consequently, the application of HA in the treatment of GR has been put forward [14]. Recent advances in smart hydrogels derived from HA paved the way for the development of stimuli-responsive biomaterials [15]. Previous studies suggested that the use of HA may enhance the clinical outcomes of root coverage but also could represent a viable option to reduce patient morbidity following the CAF technique [16,17]. A very recent systematic review and meta-analysis explored the efficacy of HA as an adjunctive in the management of GR [18]. However, due to the shortage of eligible, high-quality studies, as well as the design of the included studies limited to CAF, strong objective conclusions could not be drawn. Another systematic review and meta-analysis indicated that HA may have some slight benefits over CAF alone but added no significant benefits to SCTG [19]. By the same token, the optimal HA formulation, timing, and application technique remained unclear. The authors concluded that more research with appropriate sample sizes, diverse surgical techniques, and suitable follow-up periods should be conducted. Even though successful 6- and 12-month results were reported for MCAT in treating GR, longer-term data are still scant. Having said that, further research is needed to improve the quality of evidence regarding this topic. 

The aim of this randomized clinical trial (RCT) was to evaluate the use of MCAT and SCTG with or without cross-linked HA for the treatment of multiple RT1 and RT2 gingival recessions. The present study was a 2-year follow-up evaluation of the stability of the obtained complete root coverage (CRC), MRC, KTW, gingival thickness (GT), and root esthetic score (RES). The null hypothesis was that MRC for the HA+SCTG sides would be comparable to MRC for the SCTG sides.

## 2. Materials and Methods

### 2.1. Study Design

**Figure 1:**
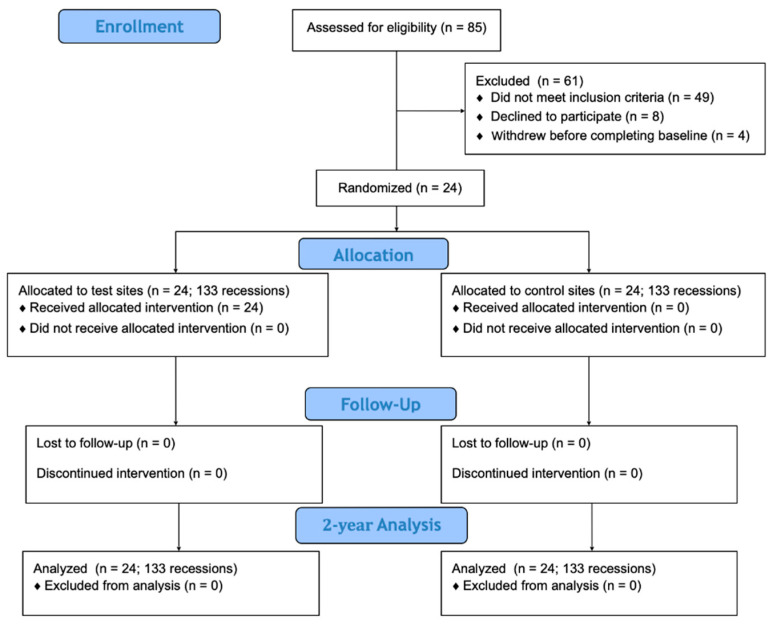
Consort diagram showing study design.

This is a 2-year follow-up of a split-mouth, double-blinded RCT [20,21]. Consequently, the evaluated patients were the same as in the previous paper, which focused on 12-month outcomes [21]. The study design is depicted in Figure 1. The trial was registered in ClinicalTrials.gov (NCT05045586, date of registration 16 September 2021) and was carried out in line with CONSORT guidelines. The study protocol respected the principles outlined in the Declaration of Helsinki and was approved by the Ethics Committee of the Medical University of Warsaw (approval number KB/119/2021, date of approval 30 July 2021). Informed consent was obtained from all participating subjects. Randomization of the 24 patients was carried out using a computer-generated randomization list by a statistician not involved in the study. The allocation was kept hidden in sealed envelopes that were opened just prior to surgery and the treatment modality was disclosed to the surgeon. Patients were blinded to the treatment allocation.

### 2.2. Exclusion and Inclusion Criteria

The following inclusion criteria were used: (1) age above 18 years old; (2) multiple GRs of type 1 (RT1) and/or type 2 (RT2) at least 1 mm height with a detectable CEJ [6]. The following criteria were used for patient exclusion: (1) full-mouth plaque score >15% and full-mouth bleeding on probing score > 15% [17]; (2) gingival recessions of type 3 (RT3); (3) systemic diseases compromising wound healing or hemostasis; (4) infectious diseases; (5) the presence of caries or restorations in the cervical area; (6) untreated periodontal diseases; (7) the intake of medication affecting periodontal status; (8) smoking; (9) drug and alcohol abuse; and (10) pregnancy or lactation.

### 2.3. Surgical Procedure and Postoperative Care

All 24 subjects were treated with the MCAT technique by the same clinician (BG), as previously described [22]. Briefly, after local anesthesia, a full-thickness flap was raised up to the muco–gingival junction (MGJ). Beyond the MGJ, a split-thickness flap was prepared. The buccal parts of the papillae were gently detached with the periosteum. A palatal SCTG was harvested following the de-epithelialized gingival graft technique [23]. In the test group, hyaluronic acid (HA, hyaDENT BG, Bioscience, Germany) was deposited onto the root surface and under the flap [24]. According to the manufacturer, 1 mL of hyaDent BG contains HA (2.0 mg), HA cross-linked (16.0 mg), sodium chloride (6.9 mg) and water (1.0 mL). Following the application of HA, the SCTG was pulled under the flap and fixed at the CEJ level with sling sutures. In the next step, HA was administered on the whole surface of the SCTG [24]. Finally, the tunnel was positioned coronally to fully cover the SCTG and the recessions with sling sutures. The control side was treated analogously, minus the application of HA.

Immediately post-surgery, patients were given analgesics and 0.12% chlorhexidine digluconate solution for 3 weeks. They were asked to avoid chewing, flossing, or brushing at the surgical site. The sutures were removed after two weeks. Patients were scheduled for follow-up appointments at 1, 3, 6, 12, and 24 months post-operatively.

### 2.4. Clinical Assessment

The following clinical parameters were registered at baseline and 6, 12, and 24 months postoperatively: gingival recession height (GRH), recession width (RW), probing pocket depth (PPD), clinical attachment level (CAL), keratinized tissue width (KTW), and gingival thickness (GT).

GRH, RW, PPD, CAL, and KTW were measured using a periodontal probe (PCP UNC 15; Hu-Friedy, Chicago, IL, USA). Measurements were rounded down to the nearest half millimeter [25]. GT was recorded using an endodontic file with a stopper positioned perpendicularly to the gingiva until the file reached the surface of alveolar bone. The distance between the stopper and the file tip was calculated using an electronic caliper with an accuracy of 0.01 (YATO YT-7201; Toya, Wrocław, Poland).

The primary outcome variable was mean root coverage (MRC) [26]. The secondary outcomes were GR reduction, CAL gain, KTW gain, GT gain, and root coverage esthetic score (RES) [26]. The RES consisted of 5 parameters: the level of the gingival margin (GM), marginal tissue contour (MCT), soft tissue texture (STT), mucogingival junction (MGJ), and gingival color (GC) [27]. The highest esthetic score was 10.

**Figure 2:**
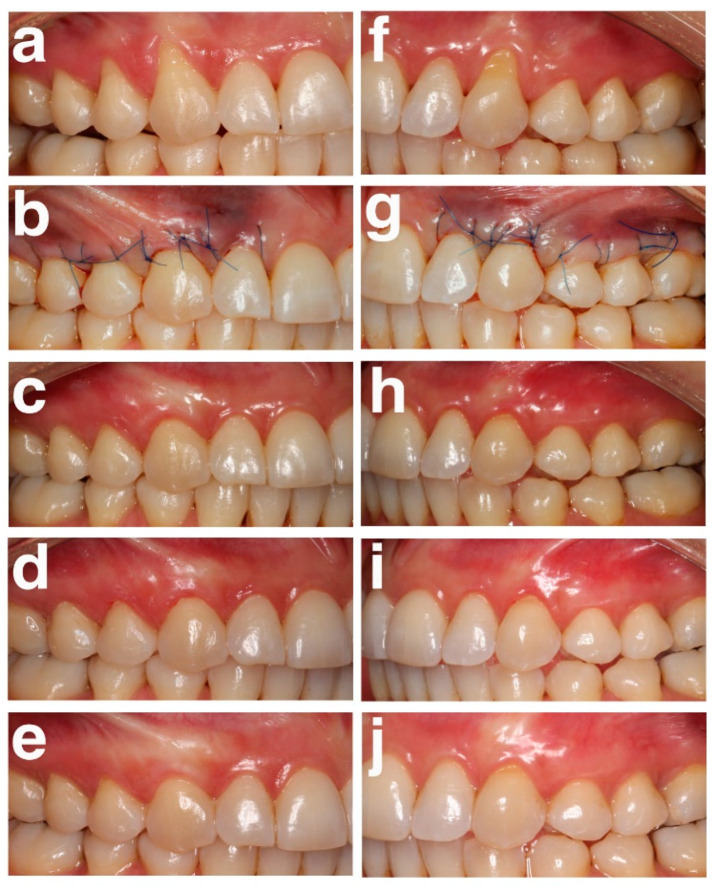
(**a**–**e**) Gingival recessions located at teeth 15–13 on the test side: (**a**) Baseline; (**b**) Immediately post-operative; (**c**) 6 months; (**d**) 12 months; (**e**) 24 months; (**f**–**j**) Gingival recessions located at teeth 23-25 on the control side: (**f**) Baseline; (**g**) Immediately post-operative; (**h**) 6 months; (**i**) 12 months; (**j**) 24 months.

All clinical and esthetic parameters were recorded by one calibrated and masked clinician (IMS). Figure 2 shows the time sequence of the test and control sides from baseline to the 24-month follow-up.

### 2.5. Examiner Calibration

The measurements were carried out by one calibrated clinician (IMS) who was unaware of the treatment allocation. A calibration exercise was performed twice within 24 h on 8 non-study patients presenting at least four contralateral GRs [8]. Calibration was accepted when ≥90% of the records were reproduced within a range of a 1.0 mm difference and exact agreement was reached in 75% of the recordings.

### 2.6. Statistical Analysis

The sample size was calculated at a significance level of 0.05 and a statistical power of 80%. The expected mean difference in MRC between the treatment groups was 8.92% and the expected standard deviation was assumed as 7.39% [28]. Based on these parameters, a sample of 12 patients per group was required. The final sample size was determined to be 24 patients due to the abundance of cases.

Statistical analysis was performed with R software v4.4.2 (R Core Team 2024). Normality of distribution for quantitative variables was assessed and confirmed using the Shapiro–Wilk test. Comparisons between the test and control sides at the same time points were evaluated using the *t*-test for independent variables. The changes in time were compared by one-way analysis of variance with the Tukey post hoc test separately for the test and control group. The analyzed outcomes were calculated as follows: (1) MRC = GR0 − GR24/GR0 × 100%; (2) GR reduction = GR0 − GR24; (3) CAL gain = CAL0 − CAL24; (4) KTW gain = KTW24 − KTW0; and (5) GT gain = GT24 − GT0. Significance was set at *p* ≤ 0.05.

## 3. Results

Twenty four patients (19 females and 5 males aged between 19 and 50, with a mean age of 32.54 ± 6.67 years) entered the study. All patients were assessed after 2 years. Two hundred and sixty-six GRs (210 in the maxilla and 56 in the mandible) were available for analysis. In the test group, 59 teeth exhibited RT1 and 74 teeth showed RT2. In the control group, 54 teeth exhibited RT1, while 79 teeth had RT2. No statistically significant differences at baseline between the two groups were noted (Table 1). No adverse events were observed during the follow-up period. Full-mouth plaque score and full-mouth bleeding on probing score were managed at or below 15% throughout the study.

**Figure 3:**
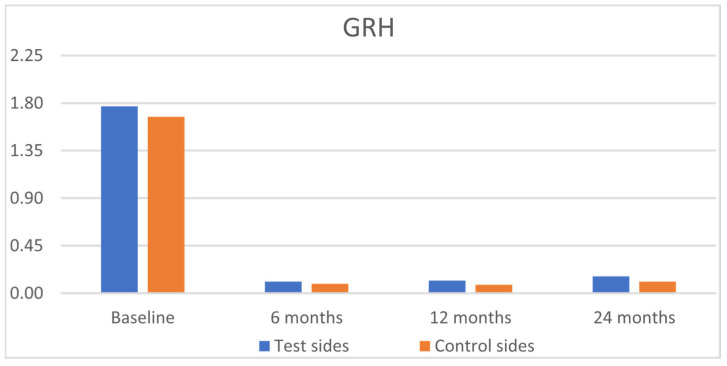
Comparison of the test sides and the control sides for gingival recession height (GRH) at baseline and 6, 12, and 24 months after surgery.

**Figure 4:**
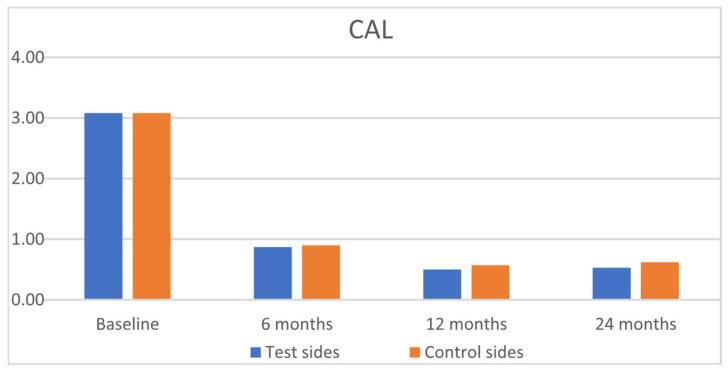
Comparison of the test sides and the control sides for clinical attachment level (CAL) at baseline and 6, 12, and 24 months after surgery.

**Figure 5:**
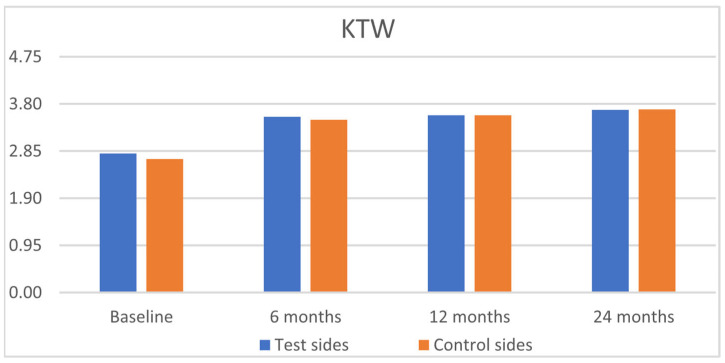
Comparison of the test sides and the control sides for keratinized tissue width (KTW) at baseline and 6, 12, and 24 months after surgery.

**Figure 6:**
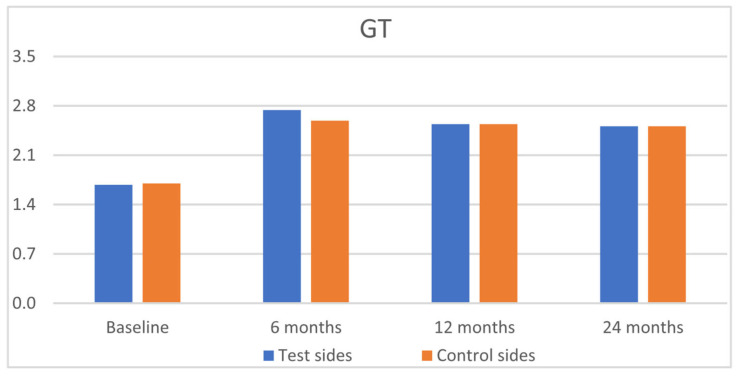
Comparison of the test sides and the control sides for gingival thickness (GT) at baseline and 6, 12, and 24 months after surgery.

The clinical results are outlined in Table 2. Statistically significant improvements were observed for GRH, RW, CAL, and GT, for both groups from baseline to 6, 12, and 24 months, with insignificant intergroup differences (Figure 3, Figure 4, Figure 5 and Figure 6). PPD and KTW did not change in any of the groups. No statistically significant differences were observed between the 6-month outcomes and both 12- and 24-month outcomes within groups.

**Figure 7:**
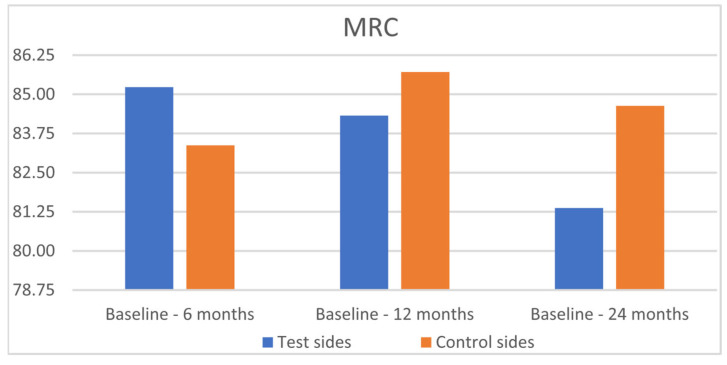
Comparison of the test sides and the control sides for mean root coverage (MRC) at 6, 12, and 24 months after surgery.

**Figure 8:**
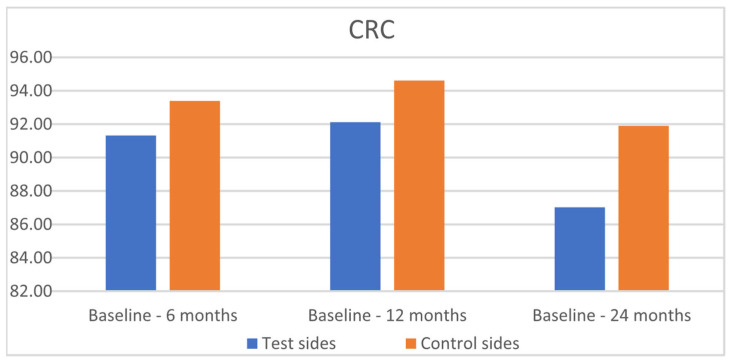
Comparison of the test sides and the control sides for complete root coverage (CRC) at 6, 12, and 24 months after surgery.

After 24 months, MRC was 81.37% for the SCTG+HA group and 84.63% for the SCTG group (*p* = 0.8192) (Table 3, Figure 7). CRC was achieved in 87.02% and 91.90% of GRs, respectively (*p* = 0.9433) (Figure 8). GR reduction was 1.66 ± 1.06 mm and 1.59 ± 1.15 mm for the test and control sides after 6 months, 1.65 ± 1.09 mm and 1.59 ± 1.15 mm after 12 months, and 1.61 ± 1.10 mm and 1.57 ± 1.15 mm after 24 months. GT gain was 1.00 ± 0.09 mm and 0.80 ± 0.98 mm for the test and control sides after 6 months, 0.81 ± 0.79 mm and 0.77 ± 0.74 mm after 12 months, and 0.78 ± 0.93 mm and 0.75 ± 0.92 mm after 24 months, respectively.

**Figure 9:**
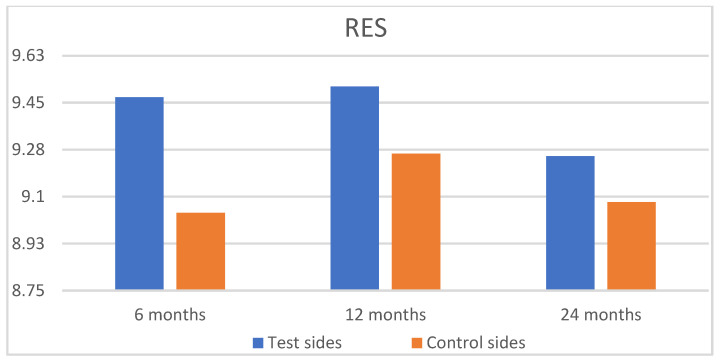
Comparison of the test sides and the control sides for root coverage esthetic score (RES) at 6, 12, and 24 months after surgery.

**Figure 10:**
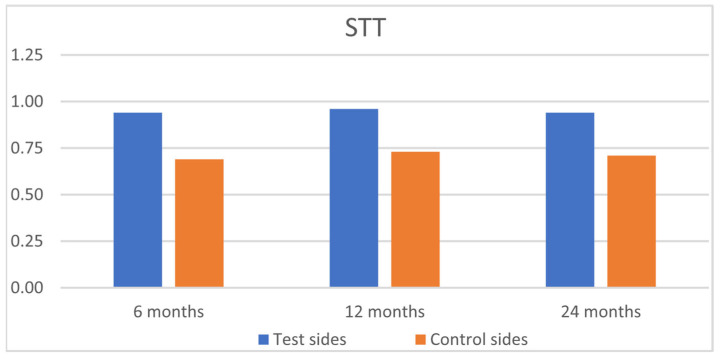
Comparison of the test sides and the control sides for soft tissue texture (STT) at 6, 12, and 24 months after surgery.

At 24 months, both groups achieved high RES scores: 9.25 for the SCTG+HA group and 9.08 for the SCTG group, respectively (Table 4, Figure 9). However, this difference was not statistically significant (*p* = 0.9704). The application of HA led to a significantly higher value for STT (0.94 for the test group vs. 0.71 for the control group) at 24 months post-operatively, respectively (Figure 10).

## 4. Discussion

The present 2-year follow-up of RCT evaluated the benefits of adjunctive HA application in combination with MCAT + SCTG in RT1 and RT2 multiple GRs treatment and compared it with MCAT + SCTG alone. The obtained outcomes showed that the test (MCAT + SCTG + HA) and control (MCAT + SCTG) procedures resulted in consistent improvements in all assessed primary and secondary outcome variables over a period of 24 months, but no statistically significant differences were observed between the groups at any time point. The obtained improvements were stable from 6 months to 24 months. Quite interestingly, no significant changes were noted in any evaluated parameters from 6 months to 24 months. The adjunctive use of HA did not influence the clinical results. The results reported in the present RCT are comparable to those of a recent systematic review and meta-analysis [9]. The only significant intergroup difference was observed in the soft tissue texture, in favor of the test group (HA). More homogenous STT in test sides was observed consistently at 6, 12, and 24 months after surgery. Based on these results, HA might be used as an adjunct to MCAT + SCTG in cases of high esthetic demand. As far as we know, there have been limited clinical application studies evaluating HA + MCAT and still no other RCT has been published so far. Consequently, a direct comparison of the results of this study with other studies is impossible.

A recent systematic review and meta-analysis indicated that local application of hyaluronic acid led to a relative root coverage (RRC) difference of 7.49% (*p* = 0.42), favoring the HA + CAF group compared to CAF alone, although statistical significance was not reached [18]. MRC varied from 58.4 ± 8.8% to 93.8 ± 13.0% for the test group and 48.1 ± 13.4% to 73.1 ± 20.8% for the control group. Two out of the three included RCTs reported better results in terms of MRC, supporting the hypothesis that HA may enhance clinical outcomes [14,17]. CRC was achieved in 80% of the HA + CAF group in contrast to 33.3% of the control group (*p* < 0.05) [17]. However, the statistical heterogeneity was high (I^2^ = 80%), mostly due to different HA formulations and follow-up timeframes. Kumar et al. applied Hyaluron gel (0.2% HA, Gengigel, Ricerfarma Pharmaceuticals, Milan, Italy) [14]. Pilloni et al. used a cross-linked high-concentrated HA gel (2 mg/mL HA, hyaDENT BG, Bioscience, Germany) [17]. The follow-up periods lasted from 6 to 18 months [14,15,16,17]. Since the abovementioned RCTs employed CAF as a flap design, it is debatable whether it would be reasonable to compare our results with the latter studies.

The clinical evidence for the adjunctive application of HA to MCAT + SCTG is still scant. So far, only two case series studies concentrated on HA in combination with SCTG and MCAT or laterally closed tunnel (LCT) in single and multiple recessions [24,29]. One case series reported on 12 single GRs [29], whereas the other included 15 multiple GRs [24]. Both studies used hyaDENT HA and reported CRC in 50 and 20% of cases, respectively. Guldener et al. [29] reported an MRC of 96.09% and a KTW increase of 3.3 ± 1.6 mm (*p* < 0.0001) from baseline to follow-up. Interestingly, Lanzrein et al. [24] noted lower MRC and CRC compared to other studies, 85.1 ± 23.2% and 20%, respectively. In this respect, single GR may have better prognosis when compared to multiple GRs. In the study by Lanzrein et al. [24], the mean RES was 7.9. Due to the lack of a control group, it was impossible to draw any conclusions about the clinical benefit of an adjunct application of HA. Nevertheless, it is clear that due to the limited evidence available and the heterogeneity of the studies, well-rounded RCTs are required to clarify a potential advantage of HA in gingival recession treatment, especially in terms of different flap designs.

HA acts through two main mechanisms, firstly by binding to its receptors (CD44, RHAMM, LYVE-1, and HARE) that are present on the cell membranes of cells such as lymphocytes, inflammatory, endothelial, and connective tissue cells and secondly, through enhancing a microenvironment that facilitates optimal wound healing [30,31]. HA improves the transport of vital metabolites, thus preserving tissue homeostasis [32]. All things considered, HA and its receptors initiate various signaling pathways, regulating cell responses to a plethora of signals. Hyaluronic acid decreases tissue disintegration by activating metalloproteinase inhibitors [33]. An in vitro study of oral fibroblasts showed that a cross-linked HA boosted the expression of genes encoding type III collagen and transforming growth factor-β3, characterizing scarless wound healing [34]. As a matter of fact, HA might appear to be especially important in the early phases of wound repair, when extracellular matrix accumulation should be limited. We previously demonstrated that biopsies taken from the test sides (MCAT + SCTG + HA) had a significantly higher density of elastic fibers and a moderate increase in the number of collagen fiber when compared to the control sides (MCAT + SCTG) [21]. It was speculated that HA was likely to affect the cell populations composing SCTG and cells in the surrounding tissue of the recipient side. This could contribute to the better esthetics of soft tissue texture observed in the present RCT. Nevertheless, this finding should be interpreted cautiously as the difference might not be clinically relevant. By contrast, CAF with the additional use of enamel matrix derivative (EMD) led to periodontal regeneration with new connective tissue (cementum and bone) [35]. The use of MCAT and SCTG yielded successful 5-year results with MRC of 73.87 (test) and 75.04 (control), but the adjunct application of EMD did not influence the results [36]. A systematic review and meta-analysis concluded that the adjunctive application of EMD in the treatment of GR provided moderate-certainty evidence in favor of its use for the reduction of GR and gain in CAL at 6 and 12 months [37]. Root coverage outcomes using platelet-rich fibrin (PRF) have gained increased interest [38]. PRF as an adjunct to CAF showed statistically significant results regarding MRC, compared to CAF alone. On the other hand, no statistically significant difference was obtained when PRF was added to CAF combined with SCTG.

HA molecular weight ranges from 5 to 20,000 kDa in vivo [39]. HA of different molecular weights and concentrations exhibits distinct modes of action, resulting in different biological effects. The native molecular design is degraded quicker than the cross-linked structure stabilized by covalent bonds [40]. Other common modification methods of HA include esterification and grafting [31]. In these RCTs, a mixture of 1.6% cross-linked HA and 0.2% linear HA was used, similarly to the majority of other studies [17,24,29], while Kumar [14] employed 0.2% linear HA. In an in vitro study, it was noted that while concentrations of HA in range of 0.01–0.1% enhanced cell adhesion, migration, and multiplication, a concentration above 0.1% reduced or even inhibited these effects [41]. Consequently, HA composition and concentration may influence the outcomes of GR surgical treatment. Another important caveat is the application method. In the present RCT, HA was placed on mechanically-treated roots without prior use of ethylenediaminetetraacetic (EDTA). It should be highlighted that during the surgery, external conditions, for example saline irrigation, may alter the characteristics and potency of HA. To guarantee permanence in the wound, in our RCT, HA was deposited twice, firstly on the root surfaces before SCTG placement and secondly immediately before suturing through infiltration under the flap. Based on the available data, it is not realistic to pinpoint which formulation and application method of HA would be optimal.

Another important caveat is the follow-up period. Other studies on HA had follow-ups that varied from 18 weeks to 30 months [14,15,16,17,24,29]. It goes without saying that longer follow-up periods are required to track the stability of surgical outcomes following GR treatment. An apical relapse of the gingival margin in CAF-treated sites was reported, while a coronal improvement of the margin was observed in CAF+CTG-treated sites from the 6-month to the 5-year follow-ups [42]. It is widely accepted that the adjunctive use of SCTG facilitates stability and reduces soft tissue contraction following root coverage treatment [43]. A very recent systematic review evaluated the long-term (≥5 years) stability of the gingival margin and periodontal soft-tissue phenotype achieved following mucogingival therapy [44]. The study reported that the stability of the gingival margin position was correlated with KTW and GT at 6-12 months after treatment. Moreover, the addition of autogenous soft-tissue grafts led to a lower increase in GRH in time. It is planned to monitor the group of treated patients in this RCT for as long as possible. It will be interesting to observe longer-term outcomes and changes in clinical parameters to evaluate the stability of 2-year root coverage (MRC, CRC), KTW, GT, and RES.

The primary limitation of our study was the evaluation of a single formulation of HA, which may have led to methodological bias. Moreover, the precise HA dose was not measured and a placebo was not used. Another limitation concerns the sample size calculation. It was based on the results of an RCT in which HA was used in combination with a different method than in the current study [21]. We started this study soon after the COVID-19 pandemic, which was detrimental to medical care in general. Patient compliance was harder to predict and the treatment was irregular, which is why 24 subjects were included in the study. The enrollment of patients with multiple and symmetrical GRs may be a source of selection bias. The external validity of this study may probably be limited since all of the surgeries were carried out by a trained clinician. Despite these limitations, the current study is the first RCT to report 2-year outcomes following multiple GRs coverage using MCAT + SCTG with or without cross-linked HA. A split-mouth study design minimized the bias that may arise because of variations in healing patterns among individuals. Other strengths of the present study are the blinded, controlled, and prospective design. The data were collected by a calibrated examiner, and all of the surgeries were carried out by one experienced surgeon. However, it is of the utmost importance that future research should analyze different concentrations, delivery systems, and appropriate timing of HA application to optimize the performance of HA and to achieve more predictable outcomes.

## 5. Conclusions

The present data indicate that the use of MCAT + SCTG can be very effective in achieving stable 2-year outcomes following multiple RT1 and RT2 gingival recession treatment. The additional application of HA may improve the soft tissue texture; however, this intergroup difference may be clinically irrelevant.

## Figures and Tables

**Table 1:** Demographics of recruited patients.

Variables	Test Group (*n* = 24, 133 Recessions)	Control Group (*n* = 24, 133 Recessions)
Sex (*n*)		
Women	19	19
Men	5	5
Tooth type (*n*)		
Incisors	42	42
Canines	23	23
Premolars	45	45
Molars	23	23
Tooth position (*n*)		
Maxillary teeth	108	105
Mandibular teeth	25	28
Type of recession (*n*,%)		
RT1	59 (44%)	54 (41%)
RT2	74 (56%)	79 (59%)

**Table 2:** Clinical parameters: gingival recession height (GRH), recession width (RW), clinical attachment level (CAL), probing pocket depth (PPD), and gingival thickness (GT) at the surgical sites at baseline and after 6, 12, and 24 months (mean + standard deviation).

	Test Group (*n* = 24, 133 Recessions)						Control Group (*n* = 24, 133 Recessions)					
	GRH [mm]	RW [mm]	CAL [mm]	PPD [mm]	KTW [mm]	GT [mm]	GRH [mm]	RW [mm]	CAL [mm]	PPD [mm]	KTW [mm]	GT [mm]
Baseline												
Mean	1.77 ^a^	3.24 ^a^	3.08 ^a^	1.42 ^a^	2.80 ^a^	1.68 ^a^	1.67 ^a^	3.32 ^a^	3.08 ^a^	1.49 ^a^	2.69 ^a^	1.70 ^a^
SD	1.13	1.88	1.28	0.54	1.38	0.72	1.12	1.82	1.22	0.57	1.28	0.75
6 months												
Mean	0.11 ^b^	0.31 ^b^	0.87 ^b^	1.40 ^a^	3.54 ^a^	2.74 ^b^	0.09 ^b^	0.29 ^b^	0.90 ^b^	1.51 ^a^	3.48 ^a^	2.59 ^b^
SD	0.41	1.11	0.80	0.54	1.46	0.80	0.38	1.11	0.82	0.57	1.32	0.68
12 months												
Mean	0.12 ^b^	0.35 ^b^	0.50 ^b^	1.42 ^a^	3.57 ^a^	2.54 ^b^	0.08 ^b^	0.25 ^b^	0.57 ^b^	1.49 ^a^	3.57 ^a^	2.54 ^b^
SD	0.48	1.29	0.85	0.53	1.49	0.74	0.39	1.09	0.80	0.54	1.26	0.67
24 months												
Mean	0.16 ^b^	0.49 ^b^	0.53 ^b^	1.35 ^a^	3.68 ^a^	2.51 ^b^	0.11 ^b^	0.33 ^b^	0.62 ^b^	1.36 ^a^	3.69 ^a^	2.51 ^b^
SD	0.50	1.41	0.88	0.49	1.52	0.72	0.41	1.15	0.82	0.53	1.22	0.64

Different lowercase letters indicate significant differences between times after surgery from Tukey’s post hoc test (*p* ≤ 0.05).

**Table 3:** Changes in mean root coverage (MRC), complete root coverage (CRC), gingival recession reduction (GR_red_), keratinized tissue width gain (KTW_gain_), and gingival thickness gain (GT_gain_) at the surgical sites after 6, 12, and 24 months (mean + standard deviation).

	Test Group (*n* = 24, 133 Recessions)					Control Group (*n* = 24, 133 Recessions)				
	MRC [%]	CRC [%]	GR_red_ [mm]	KTW_gain_ [mm]	GT_gain_ [mm]	MRC (%)	CRC (%)	GR_red_ [mm]	KTW_gain_ [mm]	GT_gain_ [mm]
Baseline—6 months										
Mean	85.23 ^a^	91.32 ^a^	1.66 ^a^	0.67 ^a^	1.00 ^a^	83.37 ^a^	93.39 ^a^	1.59 ^a^	0.65 ^a^	0.80 ^a^
SD	34.21	29.11	1.06	1.33	0.99	35.54	26.34	1.15	1.35	0.98
Baseline—12 months										
Mean	84.32 ^a^	92.12 ^a^	1.65 ^a^	0.68 ^a^	0.81 ^a^	85.71 ^a^	94.61 ^a^	1.59 ^a^	0.76 ^a^	0.77 ^a^
SD	34.46	28.14	1.09	1.40	0.79	36.43	24.71	1.14	1.36	0.74
Baseline—24 months										
Mean	81.37 ^a^	87.02 ^a^	1.61 ^a^	0.80 ^a^	0.78 ^a^	84.63 ^a^	91.90 ^a^	1.57 ^a^	0.88 ^a^	0.75 ^a^
SD	37.17	34.73	1.10	1.50	0.93	35.33	29.85	1.15	1.40	0.92

Different lowercase letters indicate significant differences between times after surgery from Tukey’s post hoc test (*p* ≤ 0.05).

**Table 4:** Esthetic parameters: gingival margin (GM), marginal tissue contour (MTC), soft tissue texture (STT), mucogingival junction alignment (MGJ), gingival color (GC), and root coverage esthetic score (RES) at the surgical sites after 6, 12 and 24 months [mean, standard deviation (SD)].

	Test Group (*n* = 24, 133 Recessions)						Control Group (*n* = 24, 133 Recessions)					
	GM	MTC	STT	MGJ	GC	RES	GM	MTC	STT	MGJ	GC	RES
6 months												
Mean	5.73 ^Aa^	0.88 ^Aa^	0.94 ^Aa^	0.92 ^Aa^	0.98 ^Aa^	9.47 ^Aa^	5.70 ^Aa^	0.83 ^Aa^	0.69 ^Ba^	0.86 ^Aa^	0.95 ^Aa^	9.04 ^Aa^
SD	0.87	0.32	0.23	0.28	0.16	1.00	1.12	0.38	0.46	0.35	0.22	1.00
12 months												
Mean	5.75 ^Aa^	0.90 ^Aa^	0.96 ^Aa^	0.92 ^Aa^	0.98 ^Aa^	9.51 ^Aa^	5.78 ^Aa^	0.87 ^Aa^	0.73 ^Ba^	0.89 ^Aa^	0.98 ^Aa^	9.26 ^Aa^
SD	0.83	0.30	0.20	0.28	0.14	1.01	0.87	0.34	0.22	0.31	0.13	1.10
24 months												
Mean	5.58 ^Aa^	0.87 ^Aa^	0.94 ^Aa^	0.87 ^Aa^	0.98 ^Aa^	9.25 ^Aa^	5.69 ^Aa^	0.84 ^Aa^	0.71 ^Ba^	0.86 ^Aa^	0.98 ^Aa^	9.08 ^Aa^
SD	1.04	0.34	0.23	0.34	0.13	1.24	1.00	0.37	0.46	0.35	0.13	1.22

Different lowercase letters indicate significant differences between time after surgery from Tukey’s post hoc test (*p* ≤ 0.05). Different capital letters indicate significant differences between test and control group from *t* test (*p* ≤ 0.05). No significant differences were found between the test and control groups from the *t*-test (*p* > 005).

## Data Availability

The raw data supporting the conclusions of this article will be made available by the corresponding author on request.
